# Laser-Induced Plasmonic Nanobubbles and Microbubbles in Gold Nanorod Colloidal Solution

**DOI:** 10.3390/nano12071154

**Published:** 2022-03-31

**Authors:** Shang-Yang Yu, Chang-Hsuan Tu, Jiunn-Woei Liaw, Mao-Kuen Kuo

**Affiliations:** 1 Department of Mechanical Engineering, Chang Gung University, 259 Wen-Hwa 1st Rd., Taoyuan City 333323, Taiwan; stcharliedavid@hotmail.com; 2 Institute of Applied Mechanics, National Taiwan University, 1 Sec. 4, Roosevelt Rd., Taipei City 106216, Taiwan; doonew9648@outlook.com; 3 Department of Mechanical Engineering, Ming Chi University of Technology, 84 Gungjuan Rd., New Taipei City 243303, Taiwan; 4 Proton and Radiation Therapy Center, Linkou Chang Gung Memorial Hospital, 15 Wen-Hwa 1st Rd., Taoyuan City 333011, Taiwan

**Keywords:** gold nanorod, surface plasmon resonance, optical breakdown, nanobubbles, single microbubble, coalesced microbubble, splitting microbubble, photoacoustic signal, pulsed laser, photothermal effect

## Abstract

In this work, we studied the initiated plasmonic nanobubbles and the follow-up microbubble in gold nanorod (GNR) colloidal solution induced by a pulsed laser. Owing to the surface plasmon resonance (SPR)-enhanced photothermal effect of GNR, several nanobubbles are initiated at the beginning of illumination and then to trigger the optical breakdown of water at the focal spot of a laser beam. Consequently, microbubble generation is facilitated; the threshold of pulsed laser energy is significantly reduced for the generation of microbubbles in water with the aid of GNRs. We used a probing He-Ne laser with a photodetector and an ultrasonic transducer to measure and investigate the dynamic formations of nanobubbles and the follow-up microbubble in GNR colloids. Two wavelengths (700 nm and 980 nm) of pulsed laser beams are used to irradiate two kinds of dilute GNR colloids with different longitudinal SPRs (718 nm and 966 nm). By characterizing the optical and photoacoustic signals, three types of microbubbles are identified: a single microbubble, a coalesced microbubble of multiple microbubbles, and a splitting microbubble. The former is caused by a single breakdown, whereas the latter two are caused by discrete and series-connected multiple breakdowns, respectively. We found that the thresholds of pulsed energy to induce different types of microbubbles are reduced as the concentration of GNRs increases, particularly when the wavelength of the laser is in the near-infrared (NIR) region and close to the SPR of GNRs. This advantage of a dilute GNR colloid facilitating the laser-induced microbubble in the NIR range of the bio-optical window could make biomedical applications available. Our study may provide an insight into the relationship between plasmonic nanobubbles and the triggered microbubbles.

## 1. Introduction

In recent decades, plasmonic nanooptics has been a fast-growing research topic [1,2,3]. When an electromagnetic wave interacts with metallic nanoparticles, free electrons perform a collective and periodic oscillation following an alternating electric field; this is called surface plasmon resonance (SPR) [3]. As a consequence, a strong absorption and scattering of light by metallic nanoparticles take place at specific wavelengths. Due to SPR, the light scattering caused by gold nanoparticles along the light path in the medium makes the light beam visible, which is called the Faraday–Tyndall effect [4]. The wavelength selectivity of SPR is dependent on the shape, size, and permittivity of the metal nanoparticles, as well as the refractive index of the surrounding medium [5]. Among these nanoparticles, various gold nanoparticles (GNPs) have drawn a lot of attention [5,6,7,8]. For example, the wavelength of the longitudinal SPR is red-shifted as the aspect ratio (AR) of a gold nanorod (GNR) increases [9,10,11,12]. If the SPR of GNRs is within the near-infrared (NIR) region, they are particularly useful for biomedical applications [13]. Recently, a variety of applications of GNP have been developed based on the plasmonic photothermal, optomechanical, and photochemical effects [14,15,16]. An important application of GNP is to utilize the plasmon-enhanced photothermal effect for generating plasmonic nanobubbles [17,18,19,20,21,22,23,24,25]. Due to the SPR of GNP, nanobubbles can be induced by the illumination of a pulsed or CW laser; the light energy absorbed by GNP is converted into heat to evaporate the water around GNP and then to generate nanobubbles [24,25,26,27,28,29]. On the other hand, owing to the strong absorption and scattering of light by SPR of GNP/GNR, the defocusing and energy decrease of a focused light beam at the focal spot occur in GNP/GNR colloidal solution [4,30]. Hence, these light-scattering and absorption effects of SPR of GNP/GNR play crucial roles in laser-induced plasmonic nanobubbles and even microbubbles in GNP/GNR colloidal solution [4,30].

In addition, the dynamic behaviors of transient bubbles caused by dielectric breakdown (an avalanche ionization) in water have been extensively studied by using high-speed cameras in recent decades [31,32,33,34]. For example, multiple oscillations (cycles) of a transient bubble were investigated; each cycle includes growing, collapse, and rebound stages. Bubble cavitation is an important issue of industrial applications. In principle, the Rayleigh–Plesset model can be used to estimate the maximum radius of a transient bubble according to its lifetime [4,34]. In order to study the dynamic formation of bubbles in detail, several techniques were developed to induce transient bubbles, e.g., electric spark and pulsed laser [31,35,36,37,38,39]. Recently, the use of a focused pulsed laser to induce optical breakdown for the study of the dynamic formation of transient bubbles has been developed due to its convenience and repeatability [33,34,35,36,37,38,39,40,41,42,43,44]. Summarizing previous studies, various types of bubbles are found: a single bubble, coalescence of multiple bubbles, bubble splitting, etc. [31,32,33,34]. These different types of induced bubbles depend on the single breakdown or multiple breakdowns, related to the plasma length of the excitation laser combined with an objective lens [38]. In addition to high-speed cameras, two other measurement techniques were developed to probe the dynamic behaviors of transient bubbles: the ultrasonic method to detect the shockwaves of the initiation and collapse of a bubble and the light-scattering method to detect the intensity of probing laser beam blocked by the projected area of a bubble [30,34]. Throughout this paper, these two techniques are used to analyze the behavior of laser-induced microbubbles. In particular, the latter is utilized to detect the induced nanobubbles at the early stage of laser shining in GNR colloid.

**Figure 1:**
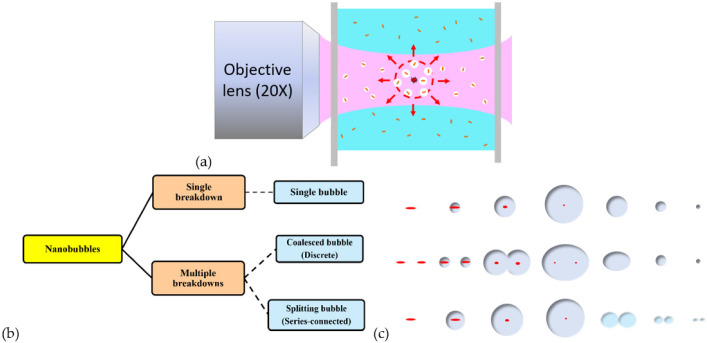
(**a**) Schematics of the formations of the initiated nanobubbles due to SPRs of GNR and the follow-up microbubble induced at the focal point of a pulsed laser beam due to optical breakdown. (**b**) Three types of microbubbles (a single microbubble, coalesced microbubbles, and splitting microbubbles) are caused by single breakdown, discrete breakdowns, or series-connected multiple breakdowns, respectively. (**c**) Configurations of three types of microbubble formation.

In this paper, we aim to study the relationship of initiated plasmonic nanobubbles and the follow-up microbubbles in GNR colloid induced by a nanosecond pulsed laser, as shown in Figure 1a. The nanobubbles are caused by the plasmon-enhanced photothermal effect of GNRs; individual GNR is a nucleus to absorb the pulsed energy of the laser for forming a nanobubble. Subsequently, one or several nanobubbles perform as sparks to trigger the optical breakdown, which is an avalanche ionization in water, at the focal spot of the laser beam. As a result, a variety of microbubbles are facilitated to be generated by a single breakdown or multiple breakdowns. Throughout this paper, three types of microbubbles (a single microbubble, coalesced microbubbles, and splitting microbubbles) are measured and classified according to the features of the signals of two sensors, a photodetector (PD) and an ultrasonic transducer (UT). These microbubbles are caused by a single breakdown, discrete breakdowns, or series-connected multiple breakdowns, respectively (Figure 1b). In the following, the effects of the concentration of GNR and pulse energy will be discussed. In addition, the on/off resonance effect on the generation of these microbubbles will be investigated by using two wavelengths (700 nm and 980 nm) of a pulsed laser to irradiate two kinds of GNR colloids with different SPRs (718 nm and 966 nm). The former wavelength is red light, and the latter is NIR light.

## 2. Method and Experimental Setup

**Figure 2:**
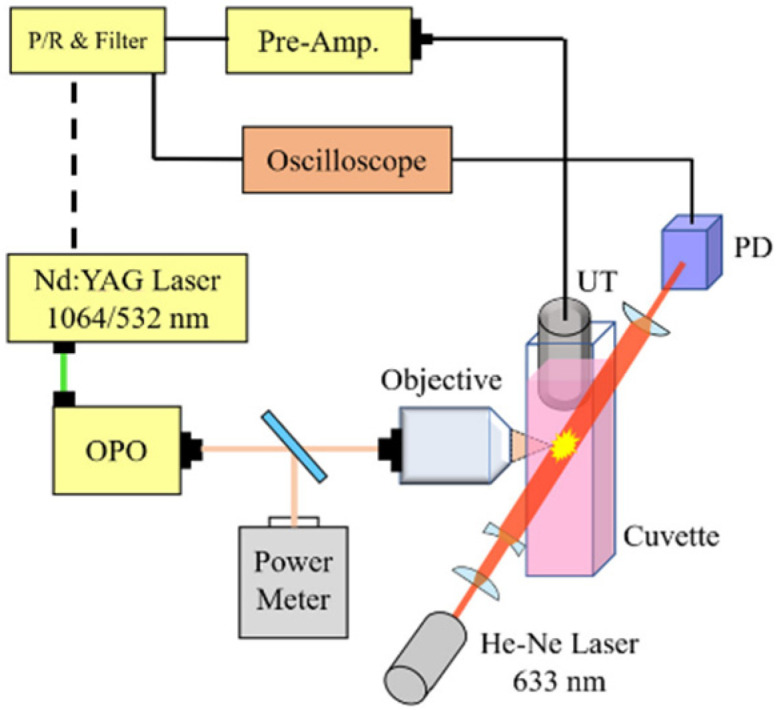
Configuration of a pulsed laser for inducing nanobubbles and microbubbles in a quartz cuvette containing GNR colloid and optical/photoacoustic measurement system. A pulsed laser of Nd:YAG with an OPO output a pulsed light with tunable wavelength (6750–1000 nm). A probing laser system (He-Ne laser and PD) for the measurement of the light-scattering signal and UT with an amplifier for the photoacoustic signal of the dynamic formation of nanobubbles and microbubbles. Three orthogonal axes (pulsed laser beam, probing laser beam, and UT) intersect at the focal point of the pulsed laser and UT.

The configuration of a pulsed laser with tunable wavelength and a measurement system is shown in Figure 2a [30]. A Q-switched nanosecond pulsed laser of Nd:YAG (SureliteII-10, Continuum, Milpitas, CA, USA) of 532 nm with a tunable optical parametric oscillator (OPO) (wavelength range: 675 to 1000 nm) is operated in a single shot mode to generate plasmonic nanobubbles and microbubbles in GNR colloids, contained in a cuvette. The duration time of each pulse is 6–10 ns. The measurement system, including a probing He-Ne laser with a photodetector (PD) and an ultrasonic transducer (UT), is utilized for measuring the light-scattering and photoacoustic signals of the dynamic formation of nanobubbles and microbubbles. The PD (ET-2030, EOT, Traverse, MI, USA) is a biased silicon detector with a cutoff frequency >200 MHz and rise/fall time of 1.5 ns. The optical axis of the He-Ne laser beam through the focal spot of the pulsed laser beam is perpendicular to that of the pulsed laser. Additionally, the axis of the ultrasonic transducer is perpendicular to these two axes; three orthogonal axes intersect at the focal point of pulsed laser and UT (V324-SM, Olympus, Tokyo, Japan). The focal length of UT is larger than 1/2 inches with a bandwidth of 25 MHz. The time of flight of ultrasonic signal from the focal spot to UT is 9.2 μs, according to our calibration. Here, the sound speed in water is 1500 m/s. This is to say that the time delay between the PD signal and UT signal is 9.2 μs; this feature is useful for us to identify the event of microbubble formation and collapse from both signals in the time domain. An ultrasound receiver (DPR300, JSR, Pittsford, NY, USA) is used for the amplification and filtering of the UT signal. A beam splitter is used to split 70% of laser energy (transmission) to an objective lens and 30% (reflection) to a power meter (NOVA II, Ophir, Jerusalem, Israel). Two long-working-distance objective lenses of 20X (NA: 0.4), LMH-20X-532 and LMH-20X-1064 (Thorlabs, Newton, NJ, USA) are used for the light in wavelength range of [450, 700] nm and [900, 1200] nm, respectively. The focal spot size of the objective lens (LMH-20X-532, Thorlabs, Newton, NJ, USA), defined by 1.83 * λ/(2 * NA), is 1.2 μm at λ = 532 nm, and is 1.6 μm at *λ* = 700 nm. The focal spot size of another objective lens (LMH-20X-1064, Thorlabs, Newton, NJ, USA) is 2.4 μm at *λ* = 1064 nm, and is 2.24 μm at *λ* = 980 nm. The Rayleigh length (*Z_R_*) of a focused Gaussian beam is $Z_{R}=\frac{\pi n\omega_{0}^{2}}{\lambda },\;$where *λ* is the wavelength of laser, *n* is 1.33 (refractive index of water), and ω_0_ is the waist of the focused laser beam. If we use half of the spot size as the waist of the Gaussian beam, the Rayleigh length is 2.83 μm in water for *λ* = 532 nm, and is 5.65 μm for *λ* = 1064 nm. The depth of field is twice the Rayleigh length. The transmittance of the objective lens (LMH-20X-532, Thorlabs, Newton, NJ, USA) operated at 700 nm is 62%, and the transmittance of the objective lens (LMH-20X-1064, Thorlabs, Newton, NJ, USA) at 980 nm is 89% (Supplementary Figure S1). In our study, the pulse energy for inducing microbubbles is calibrated by the ratio of reflection to transmission of the beam splitter and the transmittance of the objective lens. Additionally, a 12-bit digital storage oscilloscope (InfiniiVision MSOX6004A, Keysight Technologies, Santa Rosa, CA, USA) with bandwidth of 4 GHz and maximum sampling rate of 20 GHz is used for data acquisition. The sampling rate we used for optical and photoacoustic signals is 250 MHz.

The maximum plasma length *Z_max_* of a focused laser beam via an objective lens is defined by Equation (1),

(1) $$Z_{max}=\frac{\lambda }{\pi \left(tan^{2}\theta /2\right)}\sqrt{\beta -1}$$

where *λ* is the laser’s wavelength, *θ* is the focusing angle, and *β = E_L_⁄E_th_* is the normalized laser pulse energy. *E_L_* is the energy of the laser and *E_th_* the breakdown threshold [38]. In addition, we can estimate the maximum size of a transient nanobubble or a microbubble during its dynamic growth by the measured lifetime. Theoretically, the maximum radius, *R_max_*, of a single bubble in solution related to its lifetime *T* is given by Rayleigh’s model [4,34,39],

(2) $$\;R_{max}=\frac{T}{1.83}\sqrt{\frac{P_{s}-P_{v}}{\rho }}$$

where the density of water is *ρ* = 998 kg/m^3^, *P_s_* is the static pressure (100 kPa), and *P_v_* is the vapor pressure of water (3.17 kPa) [34]. Rayleigh’s model also illustrates that the maximum radius of a transient bubble is linearly proportional to ∛E,

(3) $$R_{max}=\sqrt[{3}]{\frac{3E}{4\pi P_{s}}}$$

where *E* is the total energy of bubble [4].

## 3. Material

**Figure 3:**
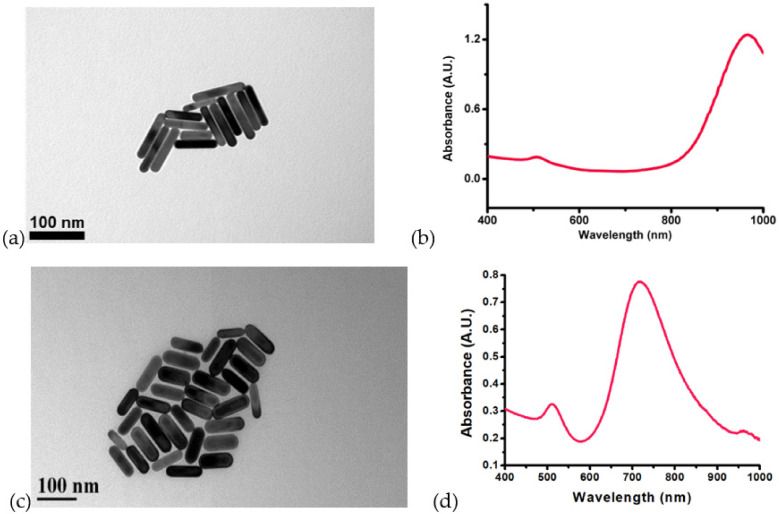
(**a**,**c**) TEM images of two kinds of GNRs with different ARs; their longitudinal SPRs are at 966 nm and 718 nm, respectively. (**b**,**d**) Absorbance spectra of the two kinds of GNRs.

We used a surfactant of cetyltrimethylammonium bromide (CTAB) to synthesize GNRs. The synthesis protocol of GNRs refers to Ref. [9]. Two kinds of GNRs were synthesized for the experiment. The detailed protocols are provided in the Supplementary Materials. Figure 3 shows the TEM (JEM-1230, JEOL, Tokyo, Japan) images of these GNRs and their absorbance spectra, respectively. The first kind of GNR has an average size in length of 89 nm and width of 15 nm with an AR of 6.1 ± 0.8; the longitudinal SPR peak is at 966 nm (Figure 3a,b). The other GNRs have an average size in length of 75 nm and width of 22 nm (AR: 3.4 ± 0.5); the longitudinal SPR peak is at 718 nm (Figure 3c,d). The two kinds of GNR colloids with various concentrations were prepared for the following irradiation of two pulsed lasers of different wavelengths (700 nm and 980 nm) with various energies to investigate the induced microbubbles. The nanoparticle (NP) density was calculated for each colloid according to the size of GNR and concentration. For example, the NP density of an 8 ppm colloid of the first kind of GNR is 2.79 × 10^10^ NP/mL, and that of the second kind of GNR is 1.61 × 10^10^ NP/mL; the average distance between adjacent GNRs is 3.3 μm and 4 μm for the former and the latter, respectively.

Although there are some residual CTABs on the surfaces of GNRs, the surface ligand has a minor effect on the photothermal effect of GNRs, as we know. In addition, the photothermal effect could cause overheating and the subsequent thermal deformation or even melting of GNRs. Consequently, the morphology change could alter the surface plasmon resonance of GNR to have different absorption efficiencies and spectra. Therefore, after a certain number of laser shots, we need to replace the colloid for the subsequent experiments to ensure the quality of GNRs.

## 4. Results and Discussion

### 4.1. Plasmonic Nanobubbles

**Figure 4:**
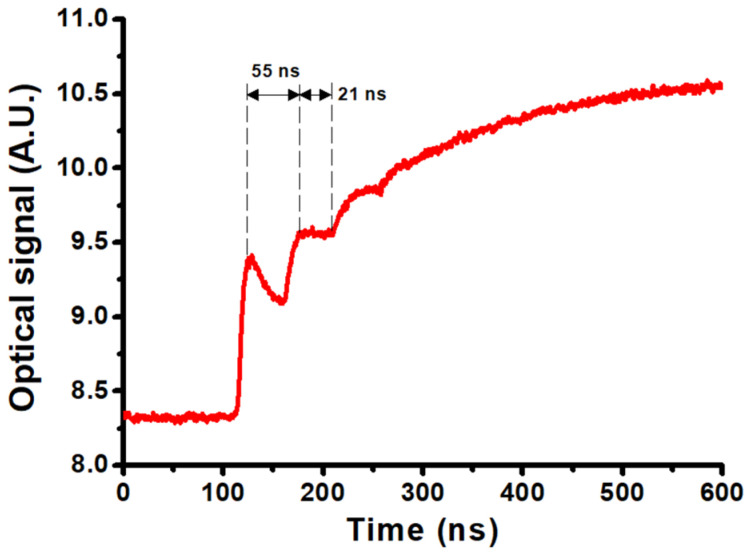
Optical signal of laser-induced plasmonic nanobubbles in 200 ppm GNRs colloid (SPR: 966 nm) irradiated by 980 nm pulsed laser of 80 mJ energy.

Plasmonic nanobubbles are generated in the very early stage of pulsed laser irradiation, where an individual nanobubble encloses a GNR. Since these nanobubbles are very small and their lifetime is very short compared to the follow-up microbubble, the light-scattering and photoacoustic signals of nanobubbles are too weak to be detected. Hence, we used a higher-energy pulsed laser without an objective lens to irradiate a high-concentration GNR colloid to increase the light-scattering signal and magnified the oscilloscope timescale to detect the nanobubbles at the very early stage. Figure 4 shows a typical optical signal of a group of nanobubbles in 200 ppm GNR colloid (SPR: 966 nm) irradiated by an 80 mJ pulsed laser of 980 nm, where the dip and shoulder in the profile indicate that the probing laser beam is blocked by a group of transient nanobubbles to suddenly reduce the intensity of light detected by PD. For this case, there are two cycles of oscillation of these nanobubbles in the focal spot; the lifetimes are 55 and 21 ns, respectively. The lifetime of the second cycle is always shorter than the first cycle, because the energy of the nanobubble is attenuated sequentially. According to the lifetime of 55 ns, the estimated maximum radius of an individual nanobubble is about 288 nm (Equation (2)). The intensity of the optical signal at the early stage of laser shining is evidence of the nanobubbles caused by the photothermal effect of GNRs within the focal spot of the pulsed laser. On the other hand, if the pulsed energy is too high, the transient temperature in GNR could be over 600 °C to melt and re-sharpen GNR [14]. Consequently, overheating may cause the change in SPR performance, which is not suitable for several shots of laser on the same GNR colloid. Therefore, a lower-energy laser pulse is used to irradiate a dilute (2 to 8 ppm) GNR colloid for the investigation of the follow-up microbubbles in our study.

### 4.2. Single Breakdown: Single Microbubble

**Figure 5:**
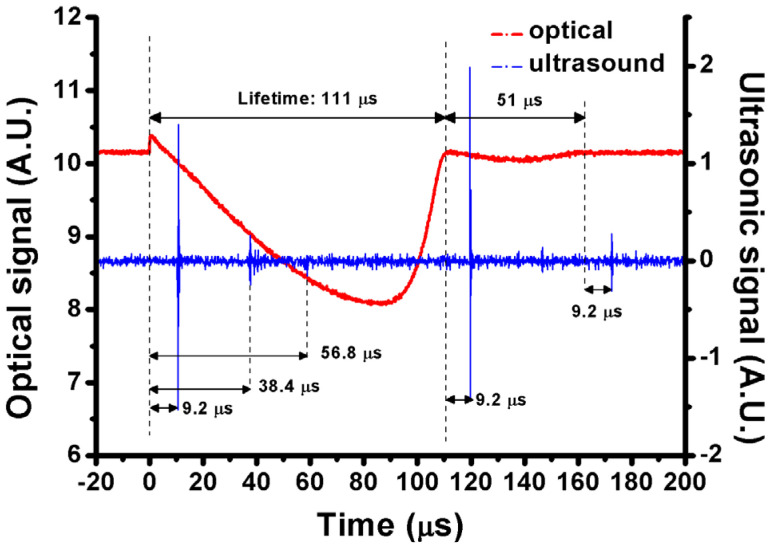
Optical (red) and photoacoustic (blue) signals of a typical single microbubble induced in 4 ppm GNR colloid with SPR of 966 nm irradiated by 980 nm pulsed laser of 8.6 mJ energy.

The PD and UT signals of a typical single microbubble are shown in Figure 5, where a 4 ppm GNR colloid with SPR of 966 nm is irradiated by a 980 nm pulsed laser of 8.6 mJ energy. The lifetimes of the first and second cycles of the microbubble are 111 and 51 μs, respectively. According to Equation (2), the maximum radius of a transient microbubble (*R_max_*) is 582 μm and 268 μm during the first and second cycles of bubble oscillation. Additionally, multiple reflections of the photoacoustic signal between UT and the bottom of the cuvette are observed.

The specific heat of gold is 0.126 J/g-K, and the melting point of GNP/GNR is about 590 °C, which is much lower than that of bulk gold (1064 °C) [14]. The latent heat of fusion for gold is 67 J/g, and the latent heat of vaporization is 1578 J/g. For a GNR, the typical volume is roughly 1.5 × 10^−17^ cm^3^ with a mass of 2.87 × 10^−16^ g, where the density is 19.32 g/cm^3^. The energy needed to melt a GNR is roughly 4 × 10^−14^ J, and the energy needed to vaporize a GNR is at least 1 × 10^−12^ J, where the boiling point is 2660 °C. However, according to Equation (3), the required energy to form a typical microbubble with a maximum radius of 200 μm is about 3.2 × 10^−6^ J; this energy is much larger than the energy to vaporize a GNR. This implies that the major energy of a microbubble generated in a dilute colloid is from the optical breakdown of water caused by a focused pulsed laser, rather than the absorbed energy by a GNR at the focal spot. In this case, some laser-induced plasma is generated inside a microbubble, accompanying the cavitation. This behavior is similar to the laser ablation [45]. On the other hand, the average energy of the measured nanobubbles with a maximum radius of 288 nm is about 10^−14^ J (Figure 4), which is less than the energy needed to melt a GNR, 4 × 10^−14^ J. For this case, we can infer that these GNRs are heated to the melting temperature by the photothermal effect, but not completely molted, and then the absorbed energy is converted into the internal energy of a nanobubble by vaporizing the surrounding water; there is no plasma generated. These tiny nanobubbles cannot trigger the optical breakdown of water, so no follow-up microbubble is induced. We thought that if the absorbed energy by a GNR from the laser beam is large enough to vaporize or even ionize GNR, the induced nanobubble might be an available spark to trigger the optical breakdown of water.

### 4.3. Discrete Multiple Breakdowns: Coalesced Microbubbles

**Figure 6:**
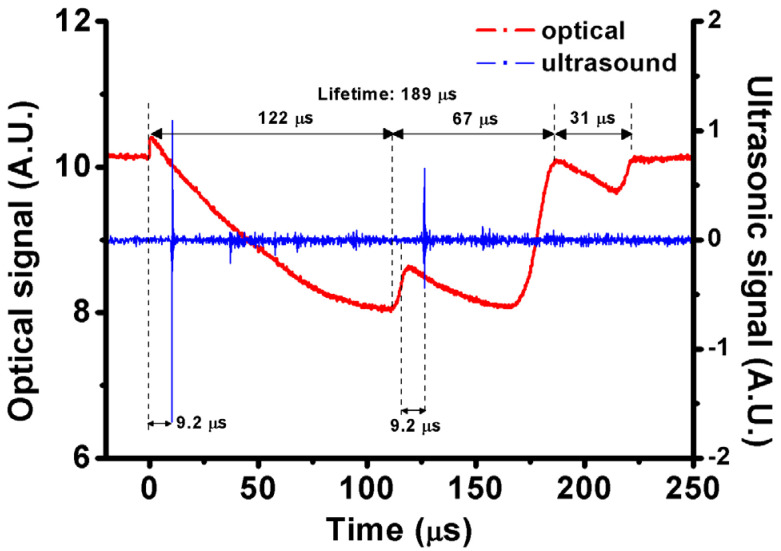
Optical (red) and photoacoustic (blue) signals of a typical coalesced microbubble of two or more microbubbles in 6 ppm GNR colloid with SPR of 966 nm induced by a 980 nm pulsed laser of 8.54 mJ energy. The lifetimes of the coalescence and re-bound cycles of microbubble are 189 and 31 μs, respectively. The coalescence induces a shockwave detected by UT.

If the plasma length of the laser beam is sufficiently large, discrete multiple breakdowns can be generated simultaneously near the focal spot. For example, as irradiated by a 980 nm pulsed laser of 8.54 mJ energy via an objective lens (LMH-20X-1064, Thorlabs, Newton, NJ, USA), two or more discrete breakdowns are induced simultaneously at the focal spot within the plasma length in a 6 ppm GNR colloid (SPR: 966 nm). Comparing the two profiles of UT and PD signals (Figure 6), we identify that the second pulse (shockwave) in UT is not an indication of bubble collapse. We infer that a coalesced microbubble of two (or more) adjacent microbubbles could happen during their expansion (growth). This may be because of discrete multiple breakdowns occurring in a sufficiently long plasma length. The dip in PD signal (122 μs after the laser shining) is an indication of the coalescence; the equivalent area of the new coalesced microbubble for blocking the probing laser beam becomes smaller than that of the two microbubbles before coalescence. After 9.2 μs, the UT would receive the shockwave signal caused by the coalescence of two or more microbubbles. Subsequently, after the collapse of the new microbubble, the second cycle is observed in the PD signal with a lifetime of 31 μs (Figure 6). The complicated process of bubble coalescence was studied by a high-speed camera in Ref. [31]. According to the lifetimes of the first cycle of 189 μs and the second cycle of 31 μs, the estimated *R_max_* values are 693 μm and 165 μm, respectively. In our experience, no such signals of UT/PD for bubble coalescence are observed by using a pulsed laser of a shorter wavelength (e.g., 700 nm) via another objective lens (LMH-20X-532, Thorlabs, Newton, NJ, USA).

### 4.4. Series-Connected Multiple Breakdowns: Splitting Microbubble

**Figure 7:**
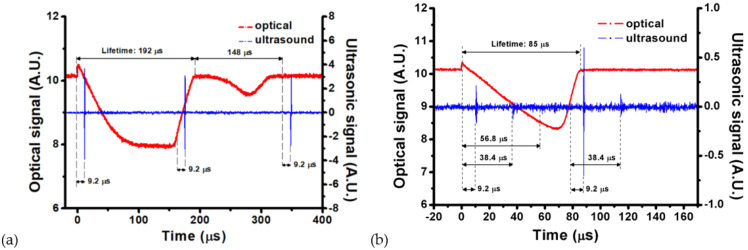
(**a**) Optical (red) and photoacoustic (blue) signals of a typical splitting microbubble in 4 ppm GNR colloid with SPR of 718 nm induced by a 700 nm pulsed laser of 17.66 mJ energy via objective lens (LMH-20X-532, Thorlabs, Newton, NJ, USA). The lifetime of the first cycle of the microbubble is 192 μs, and that of the second cycle of the split microbubbles is 148 μs. (**b**) Optical (red) and photoacoustic (blue) signals of a splitting microbubble in 6 ppm GNR colloid with SPR of 966 nm induced by a 980 nm pulsed laser of 13.04 mJ energy via objective lens (LMH-20X-1064, Thorlabs, Newton, NJ, USA).

Figure 7a,b show the other typical signals of UT and PD induced by a 700 nm pulsed laser of 17.66 mJ and a 980 nm pulsed laser of 13.04 mJ, respectively. The former is in a 4 ppm GNR colloid with SPR of 718 nm, and the latter is in a 6 ppm GNR colloid with SPR of 966 nm. For both cases, we trace the second pulse (shockwave) of the UT signal back to 9.2 μs to find that this shockwave happens before the collapse. In addition, there is no dip in the corresponding signal of PD (Figure 7a). As mentioned above (Figure 6), the dip is an indication of a coalesced microbubble. Therefore, we infer that bubble splitting occurs at this moment. For a shorter-wavelength pulsed laser (e.g., 700 nm) with higher energy via an objective lens (LMH-20X-532, Thorlabs, Newton, NJ, USA), a shorter plasma length is easily caused to facilitate the generation of line-shaped (series-connected) multiple breakdowns. As a consequence, a non-spherical microbubble expands (growths), and then a bubble splitting could happen during the collapse stage [34]. Once the bubble splitting occurs, a shockwave is generated, which can be detected by UT.

### 4.5. Threshold for Laser-Induced Microbubbles in GNR Colloid

**Figure 8:**
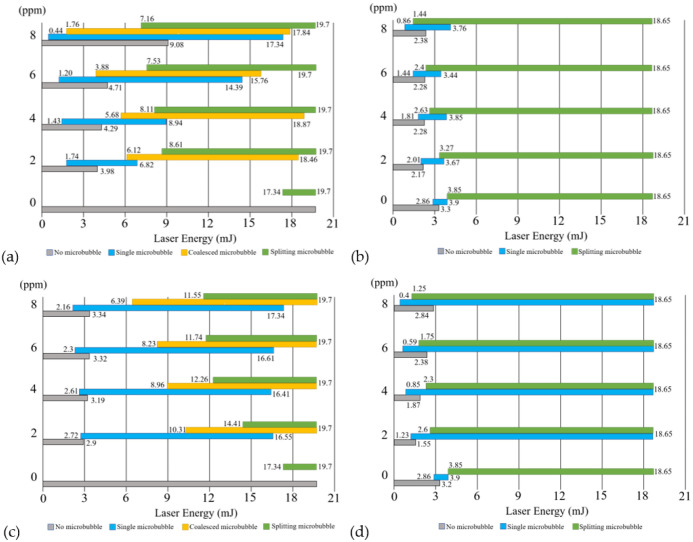
Ranges of pulsed energy to generate different types of microbubbles (single bubble, coalesced bubble, splitting bubble) in two kinds of GNR colloids with different concentrations (0, 2, 4, 6, 8 ppm). Microbubbles induced in a GNR colloid with SPR of 966 nm by pulsed lasers of (**a**) *λ* = 980 nm (on resonance) and (**b**) *λ* = 700 nm (off resonance). Microbubbles induced in a GNR colloid with SPR of 718 nm by pulsed lasers of (**c**) *λ* = 980 nm (off resonance) and (**d**) *λ* = 700 nm (on resonance).

To collect data for statistical analysis of the threshold of energy, we shot a GNR colloid of a specific concentration with a laser pulse four times, one after another. The signals of UT and PD were acquired by an oscilloscope with trigger mode. Then, we progressively increased the energy level of the laser pulse to repeat the experiment; the incremental energy level was 0.3 mJ for the 700 nm laser and 0.5 mJ for the 980 nm laser. According to the above classification of three types of microbubbles, we identify each event of microbubble based on the features of signal patterns of UT and PD for each laser shot: a single, coalesced, or spitting microbubble. The former is caused by single breakdown, and the latter two by discrete and series-connected multiple breakdowns, respectively. The charts of the distribution of pulsed energy to generate each type of microbubble in different concentrations of two GNR colloids are plotted in Figure 8. The results of the GNR colloid with SPR of 966 nm irradiated by a laser of 980 nm (on resonance) or 700 nm (off resonance) are shown in Figure 8a,b, and the results of the GNR colloid with SPR of 718 nm irradiated by a laser of 980 nm (off resonance) or 700 nm (on resonance) are shown in Figure 8c,d. In these charts, the results of deionized water are provided as reference. The thresholds of pulsed laser at various wavelengths to induce microbubbles are shown in Supplementary Figure S2. Generally, the threshold (lower bound) of pulsed energy for inducing optical breakdown is reduced with the aid of GNRs, in comparison with the reference. In addition, as the concentration of GNRs increases, the threshold (lower bound) of the pulsed energy for inducing each type of microbubble decreases. This phenomenon is significant in both types of GNR colloids irradiated by a NIR laser of *λ* = 980 nm (Figure 8a,c). Particularly when the wavelength of the laser is close to the SPR of GNRs (i.e., on resonance), the thresholds of the pulsed energy to induce a single microbubble are less than those of the off-resonance cases. We can find this behavior by comparing Figure 8a with 8b and Figure 8c with 8d. In general, a higher pulsed energy is needed to induce multiple breakdowns compared to a single microbubble. Therefore, the threshold of the required pulsed energy for inducing a coalesced or splitting microbubble is higher than that for a single microbubble.

Several previous studies using high-speed cameras have proven that the laser-induced plasma in water usually exhibits a line shape, indicating series-connected multiple breakdowns [31,32,33,34,42,43,44]. For the series-connected multiple breakdowns, a non-spherical microbubble is generated. When the non-spherical microbubble collapses, the bubble splitting easily takes place [34]. Our results also show that the probability of the occurrence of splitting microbubbles is higher than that of a single or coalesced microbubble irradiated by a higher energy pulse (Figure 8a,b). In addition, we found that a coalesced microbubble only can be induced in GNR colloid by a 980 nm laser via the objective lens of LMH-20X-1064 (Thorlabs, Newton, NJ, USA) (Figure 8a,c). In contrast, no coalesced microbubble is observed by the other objective lens (LMH-20X-532 nm, Thorlabs, Newton, NJ, USA) operated at 700 nm (Figure 8b,d). This is because the objective lens of LMH-20X-1064 (Thorlabs, Newton, NJ, USA) has a longer plasma length to facilitate the generation of discrete multiple breakdowns, leading to the coalescence of two or more microbubbles. In general, the energy threshold for a NIR laser to induce microbubbles in water is higher than that of a red-light laser, as shown in Supplementary Figure S2. By comparing the energy thresholds of lasers of *λ* = 980 nm and 700 nm to generate microbubbles in the two GNR colloids of specific concentrations, we found that with the aid of GNRs, the threshold of the former (980 nm) is significantly more reduced than the latter (700 nm). This implies that a dilute GNR colloid can significantly assist the generation of laser-induced microbubbles in the NIR region with either on resonance or off resonance (Figure 8a,c). This is an important advantage of GNRs to facilitate the laser-induced microbubble in the NIR region; the NIR light has a maximum penetration depth in tissue due to the bio-optical window. The threshold reduction of pulsed energy becomes more significant, particularly when the wavelength of laser is in the NIR range and close to the SPR of GNRs (Figure 8a,c).

### 4.6. Lifetime of Laser-Induced Microbubbles in GNR Colloid

**Figure 9:**
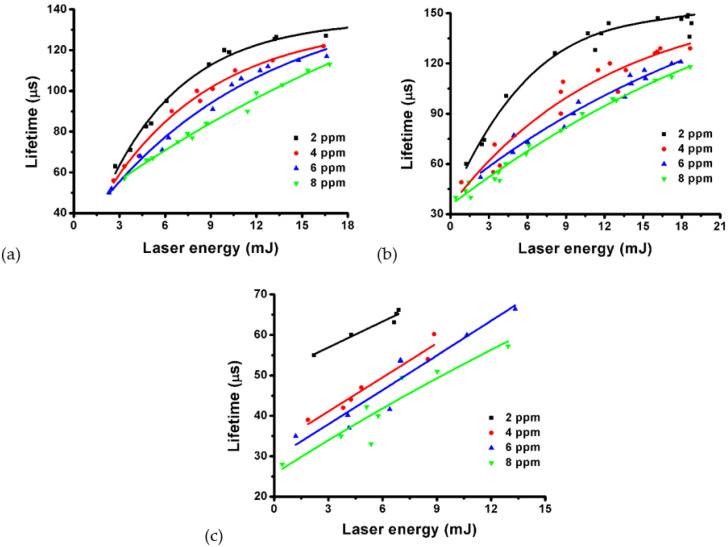
Lifetime of single microbubble versus pulsed energy of (**a**) 980 nm laser and (**b**) 700 nm laser in GNR colloids (SPR: 718 nm) of different concentrations (2, 4, 6 and 8 ppm). (**c**) The results of 980 nm laser in GNR colloids (SPR: 966 nm) of different concentrations.

We also analyzed the lifetime of single microbubble versus pulsed energy. The results of a 980 nm laser and a 700 nm laser irradiating GNR colloids (SPR: 718 nm) of different concentrations (2, 4, 6 and 8 ppm) are shown in Figure 9a,b. The results of GNR colloids (SPR: 966 nm) irradiated by a 980 nm laser is shown in Figure 9c. We find that for a fixed pulsed energy, the lifetime of a single microbubble is decreased as the concentration of GNRs is raised. This implies that the energy of an induced microbubble is less in a higher-concentration colloid. This could be due to the Faraday–Tyndall effect of GNRs, which causes strong light scattering and absorption by GNRs in the path of laser beam to reduce the energy at the focal spot [4]. The higher the concentration of GNRs, the more severe the Faraday–Tyndall effect. Therefore, the effective pulsed energy reaching the focal spot for inducing nanobubbles and the follow-up microbubbles is decreased as the concentration of GNRs increases. Even though the concentration of GNRs is a positive factor to help the threshold reduction of generating microbubbles (Figure 8), it is also a negative factor to reduce the laser intensity at the focal spot from the viewpoint of the Faraday–Tyndall effect [4]. According to Equations (2) and (3), the curve of the lifetime of a microbubble versus the pulsed energy is linearly proportional to ∛E. Figure 9 also shows that as the lifetime of a microbubble is longer than 90 μs, the curves are nonlinear in the higher-energy region, particularly for these lower-concentration colloids (2, 4, 6 ppm). If the lifetime of a microbubble is shorter than 90 μs, the relationship between lifetime and pulsed energy can be regarded as a linear approximation in the lower-energy range (Figure 9c). For 8 ppm colloids, these curves are nearly linear, as shown in Figure 9a–c. This is because the Faraday-Tyndall effect in 8 ppm colloids is more severe to reduce the laser intensity at the focal spot. In summary, in a low-concentration GNR colloid, the presence of GNRs inducing nanobubbles at the early stage of pulsed laser illumination can facilitate the optical breakdown for generating microbubbles.

## 5. Conclusions

The pulsed laser-induced plasmonic nanobubbles at an early stage and the accompanying microbubbles in dilute GNR colloids were studied. We utilized a probing He-Ne laser with a PD and UT to measure and investigate the dynamic formations of nanobubbles and microbubbles in two kinds of GNR colloids with different longitudinal SPRs (718 nm and 966 nm). The plasmon-enhanced photothermal effect of GNRs initiates several nanobubbles and then facilitates the optical breakdown of water at the focal spot of the laser beam. Along with a single microbubble caused by a single breakdown, we observed the occurrence of coalesced and splitting microbubbles caused by discrete and series-connected multiple breakdowns, respectively, according to the patterns of optical and photoacoustic signals of microbubble. The different optical breakdowns are dependent on the wavelength and plasma length of the objective lens. Our results show that as the concentration of GNRs increases, the thresholds of pulsed energy to induce different types of microbubble are reduced more, with the aid of GNRs. Particularly, when the laser’s wavelength is in the NIR region and close to the SPR of GNRs (i.e., on resonance), the threshold reduction becomes more significant due to the plasmon-enhanced photothermal effect. The advantage of using a dilute GNR colloid to facilitate the laser-induced microbubble in the NIR range of the bio-optical window could make biomedical applications available. Our study may pave the way for using NIR pulsed lasers to induce plasmonic nanobubbles for triggering microbubbles in GNP/GNR colloids in biomedical applications [4,30,44,46].

## Data Availability

Not applicable.

## Supplementary Materials

The following supporting information can be downloaded at: https://www.mdpi.com/article/10.3390/nano12071154/s1, Figure S1: Transmittances of two objective lenses (Thorlabs). (a) LMH-20X-532 and (b) LMH-20X-1064 versus wavelength. Table S1: Properties of objective lenses. Figure S2: The minimum (threshold) pulsed energy of two objective lenses (LMH-20X-532 and LMH-20X-1064) to induce optical breakdown (microbubble) in deionized water versus wavelength. References [47,48,49,50] are mentioned in Supplementary Materials.
