# Supplementary material for: Laser-Induced Plasmonic Nanobubbles and Microbubbles in Gold Nanorod Colloidal Solution
Source: Nanomaterials (Basel). 2022 Mar 31;12(7):1154. doi: 10.3390/nano12071154 (PMC9000872; doi:10.3390/nano12071154)
Supplement: Supplementary file 1 [file nanomaterials-12-01154-s001.zip › nanomaterials-1636698-supplementary.pdf]

Supplementary Materials

Laser-Induced Plasmonic Nanobubbles and Microbubbles in Gold Nanorod Colloidal Solution

Shang-Yang Yu [1], Chang-Hsuan Tu [2], Jiunn-Woei Liaw [1,3,4,*] and Mao-Kuen Kuo [2,*]

1 Department of Mechanical Engineering, Chang Gung University, 259 Wen-Hwa 1st Rd.,
Taoyuan City 333323, Taiwan; stcharliedavid@hotmail.com
2 Institute of Applied Mechanics, National Taiwan University, 1 Sec. 4, Roosevelt Rd.,
Taipei City 106216, Taiwan; doonew9648@outlook.com
3 Department of Mechanical Engineering, Ming Chi University of Technology, 84 Gungjuan Rd.,
New Taipei City 243303, Taiwan
4 Proton and Radiation Therapy Center, Linkou Chang Gung Memorial Hospital, 15 Wen-Hwa 1st Rd., Taoyuan 333011, Taiwan
* Correspondence: markliaw@mail.cgu.edu.tw (J.-W.L.); mkkuo@ntu.edu.tw (M.-K.K.)

Properties of Objective Lenses

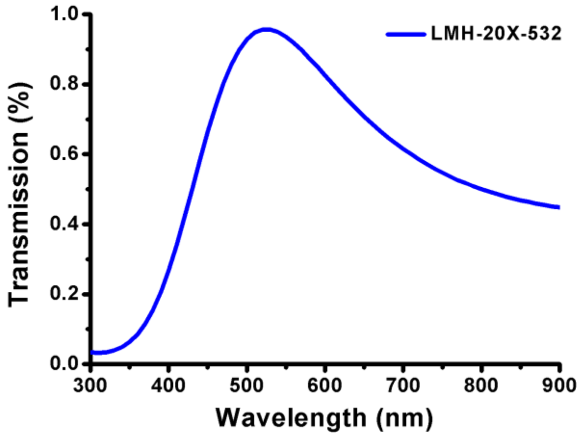

(a)

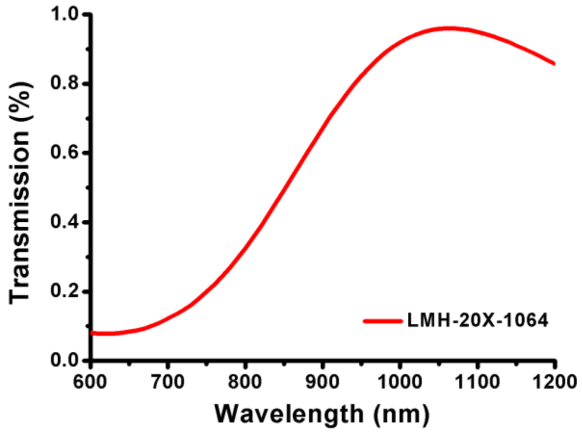

(b)

Figure S1. Transmittances of two objective lenses (Thorlabs). (**a**) LMH-20X-532 and (**b**) LMH-20X-1064 versus wavelength [1,2].

We used two kinds of 20x objective lenses (LMH-20X-532, LMH-20X-1064) for laser-induced microbubble at different wavelength, which have the best focus ability and transmittance at 532 nm and 1064 nm, respectively. The transmittances of the two objective lenses at different wavelengths are shown in **Figure S1a,b** [1,2]. For example, the transmittance of objective lens of LMH-20X-532 is 61.5% at 700 nm, and the transmittance of LMH-20X-1064 is 88.9% at 980 nm. The effective focal lengths and spot sizes of the two objective lenses in air are listed in Table S1.

Table S1. Properties of objective lenses [1,2]

	Effective Focal Length	Spot Size
LMH-20X-532	10 mm	1.2 μm
LMH-20X-1064	10 mm	2.4 μm

Threshold of Pulsed Energy of Laser-Induced Microbubble in Water

We used different-wavelength pulsed lasers via both objective lenses to induce microbubble in water. The threshold of energy for pulsed laser via the two objective lenses to induce optical breakdown (microbubble) in deionized water at different wavelengths is shown in **Figure S2**. The threshold in the region of [660, 850] nm is about 3 mJ. In contrast, the threshold is significantly raised as the wavelength of laser increase in the near-infrared region.

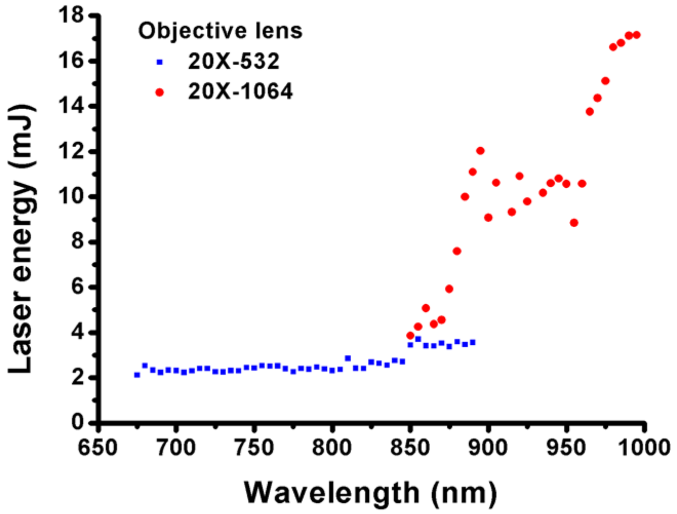

Figure S2. The minimum (threshold) pulsed energy of two objective lenses (LMH-20X-532 and LMH-20X-1064) to induce optical breakdown (microbubble) in deionized water versus wavelength.

Synthesis of GNRs

The seed-mediated method of a typical formulation by two steps was used to synthesize gold nanorods (GNRs) [3–6]. Two kinds of high-concentration GNRs with different aspect

rations (ARs) were synthesized by different protocols. We used the underpotential deposition to control AR and the size of GNRs by adding $AgNO_3$ (Sigma) and surfactant (cetyltrimethyl-ammoniumbromide; CTAB, Sigma). The detailed processes for producing the two kinds of GNRs are as follows.

(1) GNRs of Large AR

First, a gold seeds solution was prepared by mixing $HAuCl_4$ (5 mL, 0.5 mM, Aldrich) solution with CTAB (5 mL, 0.2 mM, Sigma), and then blended with ice-cold $NaBH_4$ (0.6 mL, 6 mM, Sigma) stirred at 1200 rpm. Next, a growth solution was prepared by mixing CTAB (250 mL, 10 mM) with 1.234 g NaOL at 50 °C. And then we added $AgNO_3$ (24 mL, 4 mM) and $HAuCl_4$ (12 mL, 10 mM) into the mixture stirred at 700 rpm for 90 minutes vigorously. After that, we added HCl (3.6 mL, 37 wt%) and ascorbic acid (1.25 mL, 64 mM, Sigma) successively, and then reduced the stirring rate to 400 rpm for 15 minutes. Subsequently, the mixture of the gold seeds and the growth solution was prepared, and then put in the oven at 38 °C for 12 hours. After that, the mixture was treated by centrifugation at 6000 rpm to obtain a large-AR GNR solution of high concentration.

(2) GNRs of Small AR

First, another gold seeds solution was prepared by mixing $HAuCl_4$ (25 μL, 0.1 M) solution with CTAB (10 mL, 0.1 M), and then blended with ice-cold $NaBH_4$ (0.6 mL, 10 mM) stirred at 1200 rpm. Next, another growth solution was prepared by mixing $HAuCl_4$ (48 mL, 10 mM), $AgNO_3$ (4.8 mL, 10 mM), CTAB (960 mL, 0.1 M), ascorbic acid (7.68 mL, 0.1 M) and HCl (19.2 mL, 1 M) successively. Subsequently, the mixture of the gold seeds and the growth solution was prepared, and then put in the oven at 38 °C for 12 hours. After that, the mixture was treated by centrifugation at 6000 rpm to obtain a small-AR GNR solution of high concentration.

Finally, the morphology of the two kinds of GNR solutions were measured by HR-TEM, and their concentrations were measured by ICP-AES. The AR of the former is 6.1, and that of the latter is 3.4.

References

1. Thorlabs - LMH-20X-532 High-Power MicroSpot Focusing Objective, 20X, 495 - 570 nm, NA = 0.40
2. Thorlabs - LMH-20X-1064 High-Power MicroSpot Focusing Objective, 20X, 980 - 1130 nm, NA = 0.40
3. Chang, S.S.; Shih, C.W.; Chen, C.D.; Lai, W.C.; Wang, C.R.C. The shape transition of gold nanorods. *Langmuir* **1999**, *15*, 701–709.
4. Jana, N.R.; Gearheart, L.; Murphy, C.J. Seed-mediated growth approach for shape-controlled synthesis of spheroidal and rod-like gold nanoparticles using a surfactant template. *Adv. Mater.* **2001,** *13*, 1389–1393.

5. Nikoobakht, B.; El-Sayed, M.A. Preparation and growth mechanism of gold nanorods (nrs) using seed mediated growth method. *Chem. Mater.* **2003**, *15*, 1957–1962.
6. Chen, Y.-S.; Frey, W.; Kim, S.; Homan, K.; Kruizinga, P.; Sokolov, K.; Emelianov, S. Enhanced thermal stability of silica-coated gold nanorods for photoacoustic imaging and image-guided therapy. *Opt. Express* **2010***, 18*, 8867–8878.
